# Structural characterization and immunomodulatory activity of a novel polysaccharide from *Panax notoginseng*

**DOI:** 10.3389/fphar.2023.1190233

**Published:** 2023-05-15

**Authors:** Xue-Lian Jiang, Gai-Fan Ma, Bin-Bin Zhao, Yan Meng, Lin-Lin Chen

**Affiliations:** ^1^ Key Laboratory of Traditional Chinese Medicine Resource and Compound Prescription, Ministry of Education, Hubei University of Chinese Medicine, Wuhan, China; ^2^ School of Basic Medicine, Hubei University of Chinese Medicine, Wuhan, China; ^3^ School of Pharmacy, Hubei University of Chinese Medicine, Wuhan, China

**Keywords:** panax notoginseng, polysaccharides, structural characterization, immunomodulatory activity, toll-like receptor 2

## Abstract

**Introduction:** Polysaccharides are important components of Panax notoginseng that contribute to its immunomodulatory ability. This study aimed to isolate polysaccharides from notoginseng and investigate the structural feature and potential immunomodulatory activity.

**Methods:** The polysaccharide was isolated from notoginseng by anion exchange and gel permeation chromatography. Its preliminary structure was characterized by Fourier transform infrared (FT-IR) spectroscopy, gas chromatography-mass spectrometry (GC-MS) and nuclear magnetic resonance (NMR) spectroscopy. The immunoregulatory function was further investigated in cyclophosphamide induced immunosuppressive mice, murine splenocytes and macrophages.

**Results:** A novel homogeneous polysaccharide (PNPB1) was isolated from notoginseng with the molecular weight of 9.3 × 10^5^ Da. Monosaccharide composition analysis indicated that PNPB1 consisted of Glc (88.2%), Gal (9.0%), Ara (2.4%) and trace GlcA, with the major backbone of (1→4)-linked α-Glcp, (1→6)-linked β-Glcp, and (1, 4→6)-linked β-Glcp. The polysaccharide was found to significantly enhance murine body weight, improve their thymus and spleen indices and increase the white blood cells (WBC). PNPB1 significantly enhanced splenic lymphocyte proliferation, NO and cytokine (TNF-α, IL-2, IL-10 and IFN-γ) production, as well as the phagocytosis and TLR2 expression of peritoneal macrophages, indicating potent immunoenhancement effect.

**Discussion:** These findings provide a theoretical basis for elucidating the structure and immune activity of notoginseng polysaccharides.

## Introduction

*Panax notoginseng* (Burk.) F.H. Chen, known as *Sanqi* or *Sanchi* in Chinese, is a precious medicinal plant cultivated in south-western China. The root and rhizome of *P. notoginseng* have been traditionally used as a tonic and haemostatic in East Asian countries for centuries. Notoginseng possesses various bioactive ingredients such as saponins, flavonoids and polysaccharides, exhibiting numerous pharmacological effects including haemostatic, antioxidant, hypolipidaemic, hepatoprotective, anti-tumour and immunomodulatory activities (Wang et al., 2016). Low molecular weight components, especially the dammarane-type saponins, are believed to be the principal bioactive components of notoginseng and have been well studied. The high molecular weight fraction, mainly *P. notoginseng* polysaccharides (PNP), was ignored previously and has been attracting more attention due to its immunomodulating activities. Notoginseng saponins, most of which are dammarane-type triterpene saponins, have been intensively studied in recent decades, revealing their favorable roles in the improvement of nervous, cardiovascular, endocrinic and immune functions (Peng et al., 2018). However, our attention to bioactive polysaccharides in notoginseng is relatively limited. Polysaccharides are primary classes of biomacromolecules which widely exist in animals, plants, fugus and algae. Searching for bioactive polysaccharide in natural organisms is still of great interest due to their health beneficial characteristics with low toxicity. Immunoregulation is acknowledged as the most prominent activity of polysaccharides, as evidences have been accumulating that polysaccharides distinctly affect immune cells and cytokine production associated with immune responses (Gao et al., 1996). The use of immunomodulator provides significant advantages over conventional therapies in suppressing disorders such as infection, tumorigenesis and autoimmune diseases, and the enhancement of host immune response has been recognized as a safe and effective mean to control tumor growth (Khan et al., 2019). A recent study demonstrated that crude polysaccharide of notoginseng could inhibit the growth of H22 cells and prolong the survival of tumor-bearing mice by activating CD4^+^ T-cell and elevating serum IL-2 (Li et al., 2016). Two homogeneous polysaccharide fractions obtained from notoginseng were reported to increase stress resistance and extend lifespan in *C. elegans* through the elevation of antioxidant enzyme activities (Feng et al., 2018). Nowadays, the demand of notoginseng in health product market continuously increases with the discovery of its beneficial functions. Having long been characterized as potent anti-tumor and immunomodulatory agents, notoginseng polysaccharides are believed to play an indispensable role in the tonifying action of remedy or functional foods (Wu et al., 2022). However, due to the difficulty of purification and structure elucidation, little information was available on the detailed composition and structure of the polysaccharides from notoginseng.

In the present study, a novel homogeneous polysaccharide was isolated from notoginseng by anion exchange resins and gel-filtration chromatography. The preliminary structure of polysaccharide was characterized by physicochemical property, Fourier transform infrared (FT-IR) spectroscopy, gas chromatography-mass spectrometry (GC-MS) and nuclear magnetic resonance (NMR) spectroscopy. Consequently, the immunomodulatory activities were evaluated using immunosuppressive mice, murine splenocytes and peritoneal macrophages. This work will provide chemical and bioactive information for the structure elucidation and potential application of notoginseng polysaccharides.

## Materials and methods

### Materials and chemicals

*P. notoginseng* was obtained from Panlongyunhai Pharmaceutical Co. Ltd (Chuxiong, China). DEAE Bestarose FF was purchased from Bestchrom (Shanghai, China). Sephadex G-200 was purchased from Solarbio (Beijing, China). Standard monosaccharides, trifluoroacetis acid (TFA), methyl iodide were supplied by Adama-beta Co. (Shanghai, China). Pyridine, dimethyl sulphoxide (DMSO), acetic anhydride and sodium hydride were purchased from General-Reagent Co. (Shanghai, China). Methanol (HPLC grade) was from Tedia (Fairfield, OH, United States). 1-pheny-3-methyl-5-pyrazolone (PMP) was from Macklin Biochemical Co., Ltd. (Shanghai, China). Cyclophosphamide (CTX) was provided by Ammonium acetate was from Anaqua Chemicals (Wilmington, DE, United States). All other chemicals and solvents were of analytical grade.

### Extraction and purification of polysaccharides from *P. notoginseng*

The crude polysaccharide was prepared following the ethanol subsiding method (Wang et al., 2017a). Grinded notoginseng of 100 g was first refluxed with 95% ethanol to eliminate low molecular components. After filtration, the residue was extracted 3 times with distilled water (solid-liquid ratio of 1:10, w/v) at 80°C for 2 h, and centrifuged at 9,000 *g* for 5 min, then the supernatant was combined and concentrated by rotary evaporator at 60°C under reduced pressure. The concentrated extract was precipitated by adding 4 volumes of ethanol and placed at 4°C for overnight. The resulting precipitate was dissolved in appropriate volumes of distilled water to remove protein by Sevag reagent, and then lyophilized to obtain crude polysaccharide (Sun and Liu, 2008).

The crude polysaccharide (500 mg) was redissolved in distilled water and passed through DEAE Bestarose FF column (50 × 600 mm) and eluted successively with distilled water, 0.1, 0.2, 0.3 and 0.5 M NaCl solutions at a flow rate of 2 mL/min (Li et al., 2017a). The four main fraction assigned as PNPA, PNPB, PNPC and PNPD were collected by the anthrone colorimetric method (Chen et al., 2016). The fraction of PNPB was dialyzed (MWCO 3000 Da) and lyophilized, and then PNPB of 200 mg was passed through Sephadex G-200 column (16 × 1,200 mm) with distilled water at a flow rate of 0.4 mL/min. The main fraction was collected and lyophilized to produce PNPB1.

### Homogeneity and molecular weight analysis

The homogeneity and molecular weight of PNPB1 were determined by high-performance gel permeation chromatography (HPGPC) using a Thermo Ultimate 3,000 system (Thermo Fisher Scientific Co., United States) equipped with an Alltech 2000 ES evaporative light scattering detector (ELSD) and a TSK gel G4000_PWXL_ column (7.8 × 300 mm, Tosoh Corp, Tokyo, Japan). The sample solution (1.0 mg/mL, 10 μL) was injected and eluted with distilled water at 30°C with a flow rate of 1.0 mL/min (Qiao et al., 2010). The molecular weight of PNPB1 was estimated by reference to the calibration curve established by Dextran standards of known molecular weight (80 kDa, 150 kDa, 270 kDa, 410 kDa, 670 kDa and 990 kDa).

### Monosaccharide composition analysis

The monosaccharide composition of PNPB1 was analyzed by a previously described pre-column derivatization HPLC method (Chen et al., 2017a). PNPB1 (5 mg) was sealed and hydrolyzed in 2 mL of 2.5 M TFA at 120°C for 4 h. After removing the residual THA with methanol under reduced pressure, the released monosaccharides were derivatized with PMP. The derived product was then analyzed by HPLC equipped with an Agilent Eclipse plus C_18_ column (4.6 × 100 mm, 3.5 μm) and UV detection at 245 nm according to a previously described method (Wang et al., 2018).

### Methylation analysis

PNPB1 was methylated according to a previously described method (Cai et al., 2018). The methylated products were analyzed by FT-IR spectra, in which the disappearance of the O-H absorption peak (3400 cm^−1^) indicated complete methylation (Li et al., 2017b). The methylated PNPB1 was analyzed by a GC-MS/MS system consisted of a Thermo Trace 1310 GC coupled to TSQ8000 Triple Quadrupole MS (ThermoFisher Scientific, MA, USA) equipped with a TR-5MS column (30 m × 0.25 mm × 0.25 μm). The temperature program increased from 100°C to 280°C by 5°C/min and kept at 280°C for 5 min. The injection temperature was 260°C and ion source temperature was 280°C.

### UV-Vis and FT-IR spectroscopy analysis

PNPB1 (2.0 mg) was dissolved in 1 mL of distilled water, and detected the UV-Vis absorption spectra in the wavelength rang of 190–700 nm. PNPB1 (2.0 mg) was mixed with about 200 mg of KBr and pressed into a pellet for FT-IR determination in the frequency range of 4,000–400 cm^−1^.

### NMR spectroscopy analysis

The dried PNPB1 of 40 mg were dissolved in D_2_O (1.0 mL) followed by H/D exchange reaction for three times. The ^1^H, ^13^C, HMBC and HSQC spectra of PNPB1 were recorded by Agilent DD2 600 MHz NMR spectrometer (Agilent Technologies, Santa Clara, CA, United States) with chemical shifts given in ppm.

### Scanning electron microscopy (SEM) analysis

The polysaccharide powder was coated with conductive gold, and then examined using a JSM-6510 SEM system (JEOL, Tokyo, Japan) under different magnifications.

### Immunosuppressed mice modelling and treatment

Twenty-four male BALB/c mice (6–8 weeks old, weighing 18–20 g) purchased from Liaoning Changsheng Biotechnology Co., Ltd. (Shenyang, China) were housed at a constant temperature (21°C ± 2°C) with a 12 h light-dark cycle with free access to food and water. The experimental protocols were approved by the Ethics Committee of Hubei University of Chinese Medicine. After 1 week acclimatization, the mice were randomly divided into 4 groups with 6 for each. The low and high dose treatment groups were given PNPB1 by intraperitoneal injection with the dosage of 5 and 20 mg/kg/d respectively for 14 days, whereas the mice of control and model group were treated with normal saline. The model and treatment groups were subjected to immunosuppression by intraperitoneal injection of CTX (80 mg/kg/d) at day 10, 11, 12 and 13. The weight of each animal was recorded daily throughout the experiment. Twenty-four hours after the last drug administration, the animals were subjected to carbon clearance test and then sacrificed for harvesting immune organs.

### White blood cell (WBC) and red blood cell (RBC) counting

Whole blood samples (100 μL) were collected from the retro-orbital plexus of mice 12 h after the last treatment and the WBC and RBC count of peripheral blood were analyzed using Mindray Vet2800 blood analyzer (Shenzhen, China).

### Carbon clearance test

The phagocytosis of macrophages in liver and spleen was assessed based on the scavenging efficiency of carbon particles in blood (Hamid et al., 2021). At the end of 14 days, the mice were injected with carbon ink (10 μL/g body weight) via tail vein. Blood samples were drawn from the retro-orbital vein at intervals of 2 (T1) and 6 (T2) min, 25 μL of which was mixed with 0.1% sodium carbonate solution (2 mL) and the absorbance measured at 660 nm. The carbonic particle clearances index (K) and macrophage phagocytose index (α) as well as body weight index were calculated as below:

$$Carbonic\;particle\;clearances\;index\;\left(K\right)=\frac{\log A1-\log A2}{T2-T1}$$

$$Macrophage\;phagocytose\;index\;\left(\alpha \right)=\frac{\sqrt[{3}]{K}\times weight}{Liver+spleen\;weight}$$

### Analysis of body weight and organ index

Mice were weighed and killed by cervical dislocation after carbon clearance test, and thymus and spleen were excised from the mice and weighted immediately. The thymus and spleen indexes were calculated according to the following formula:

$$Organ\;index\;\left(mg/g\right)=organ\;weight\;\left(mg\right)\;/\;body\;weight\;\left(g\right)$$

### Splenocyte proliferation assay

BALB/c mice at 8–10 weeks of age were used for obtaining splenocytes and peritoneal macrophages. Splenocytes were prepared as described by Chen et al. (2019). Cell suspension (2.5 × 10^6^ cells/mL) were seeded into the 96-well plate containing indicating concentrations of PNPB1 (5, 10, 50, 200 μg/mL) or ConA (5 μg/mL) or only RPMI-1640 medium in triplicate and then incubated for 2 days at 37°C in humidified 5% CO_2_. Cell proliferation was measured using a CCK-8 assay kit according to the manufacturer’s instructions. The ODs were measured using a Tecan Sunrise microplate reader (Tecan, Männedorf, Switzerland) at 450 nm. The proliferation of splenocytes was defined with stimulation index (SI) showed as follow: SI = sample absorbance/control absorbance.

### Determination of nitric oxide (NO) and cytokines production in splenocyte

Splenocytes (150 μL) were seeded into a 96-well plate at a density of 2.5 × 10^6^ cells/mL and incubated with PNPB1 at different concentrations (5, 10, 50, 100, and 200 μg/mL) or ConA (5 μg/mL) or RPMI-1640 medium alone. After 48-h stimulation, NO content in cell supernatant was determined by Griess reaction and the concentrations of cytokines (TNF-α, IL-2, IL-4, IL-10, IFN-γ) were measured by ELISA kits (NeoBioscience, Shenzhen, China) according to the manufacturer’s instructions.

### Neutral red phagocytosis assay of macrophages

Phagocytosis of peritoneal macrophages was measured by neutral red uptake assay as previously described (Weeks et al., 1987). Peritoneal macrophages collected from BALB/c mice were seeded at a density of 2.5 × 10^6^ cells/mL in 96-well plate with or without PNPB1 (5, 10, 50, 200 μg/mL) or LPS (1 μg/mL) and cultured for 24 h. Supernatants were removed and 100 μL of 0.1% (w/v) neutral red solution was added and incubated for 30 min. Subsequently, the cells were washed 3 times with PBS to remove excess dye and lysed in 200 μL of 50% ethanol containing 1% glacial acetic acid. The absorbance was read at 550 nm on a microplate reader.

### TLR2/4 protein assay of macrophages

In-Cell Western (ICW) is an immunofluorescence approach utilizing near infrared fluorophore-conjugated antibodies to measure individual proteins in fixed cells, which was performed to detect TLR2/4 protein level in present study using Odyssey Infrared Imaging System (LI-COR Biosciences). The In-Cell Western Kits provide detection reagents for cell-based ICW assays, which includes blocking buffer, IRDye 800CW secondary antibody for detection of a specific protein in 800 nm channel, and two fluorescent cell stains used in 700 nm channel to normalize variations in cell number. Peritoneal macrophages were collected and cultured in DMEM (containing 10% FBS) with or without PNPB1 (5, 10, 50, 200 μg/mL) or FSL (1 μg/mL) and then cultured for 24 h. After removing the medium, cells were fixed by adding 25 μL of 4% formaldehyde and incubating for 20 min, and then washed by 0.1% Triton X-100. Cells were blocked by LI-COR blocking buffer for at least 2 h at room temperature followed by incubation with primary antibodies (1:1,000 dilution, cell signaling technology, Boston, United States) and celltag 700 stand internal reference (1:800 dilution, LI-COR) solutions diluted in blocking buffer, respectively, at 4 °C overnight. After washing with 0.1% (v/v) Tween-20, cells were incubated with IRdye 800CW Goat anti-rabbit secondary antibody at 1:5,000 in blocking buffer for 2 h at room temperature, and then washed with 0.1% Tween-20 again. The plate was scanned at 700 and 800 nm channels at the same time to obtain fluorescent images and output data.

### Statistical analysis

Statistical analysis was performed by GraphPad Prism 6 (GraphPad Software, Inc., San Diego, CA, United States). Statistical significance between two groups was analyzed by Student’s t-test and multigroup comparisons were compared using one-way analysis of variance (ANOVA). Data was presented as the mean value ±SD, and *p* < 0.05 was considered statistically significant.

## Results and discussion

### Isolation and purification of polysaccharides

**FIGURE 1:**
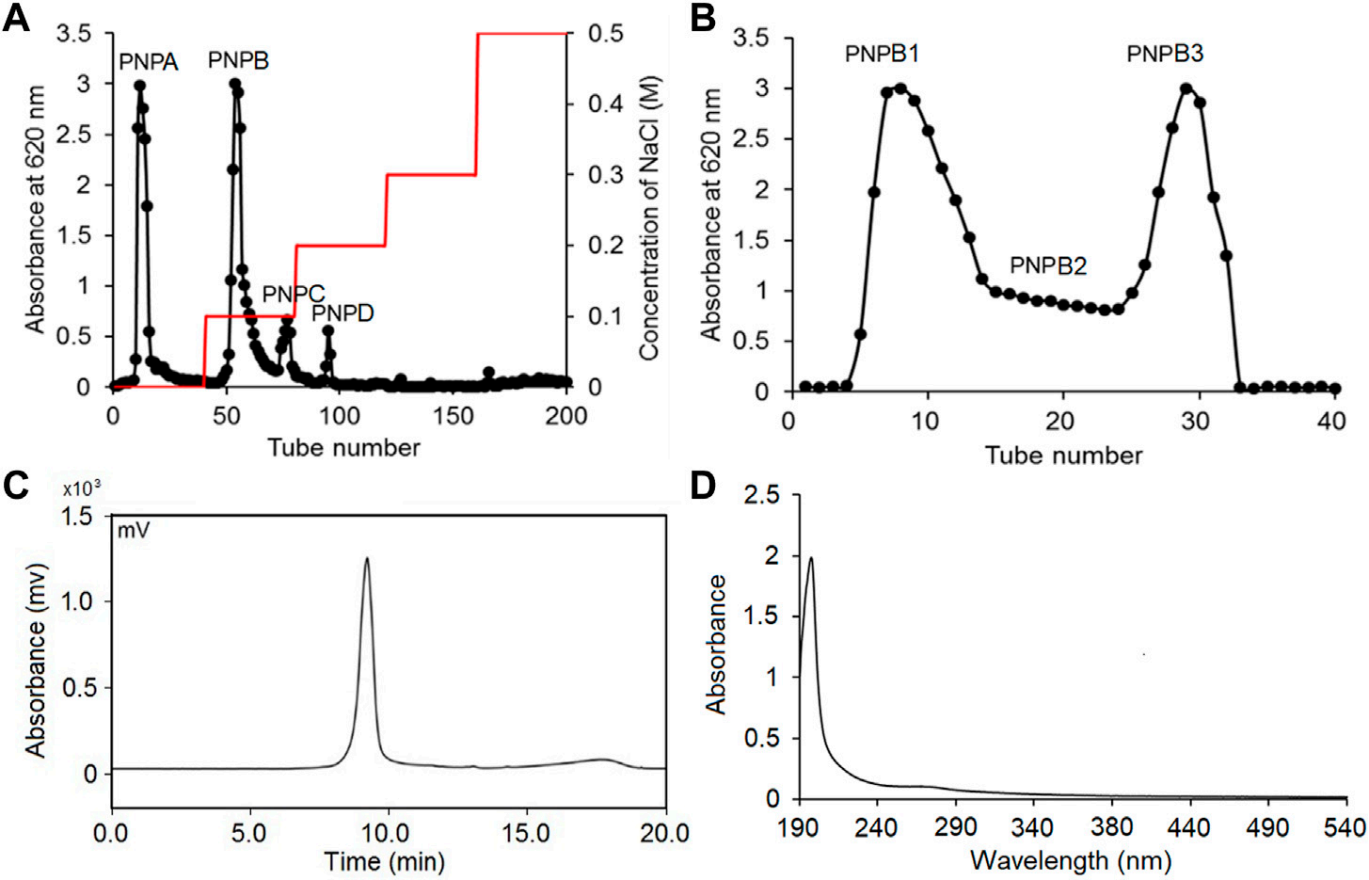
PNPB1 was extracted, purified and detected as a homogeneous polysaccharide. **(A)** The crude extracellular polysaccharides were separated by DEAE-Sepharose Fast Flow column. **(B)** Sephadex G-200 column. **(C)** GPC of PNPB1. **(D)** UV absorption of PNPB1.

The crude polysaccharides were isolated from *P. notoginseng* by hot water and ethanol precipitation and the yield was determined as 12.48% after removing protein with Sevag method. As shown in Figure 1A, two main fractions, PNPA (57%) and PNPB (24%), were eluted by DEAE Bestarose FF column chromatography with water and 0.1M NaCl solution. The fraction of PNPB was then further purified by Sephadex G-200 column chromatography to obtain two fractions of PNPB1 (54%) and PNPB2 (33%) (Figure 1B). Scanned UV spectrum showed there was no absorption peak at wavelengths of 260 and 280 nm in Figure 1D, indicating the absence of proteins and nucleic acids in PNPB1(Lu et al., 2019). Besides, PNPB1 was homogenous as it showed a single and symmetrical sharp peak in Figure 1C, with a molecular weight of 9.3 × 10^5^ Da.

### FT-IR analysis

**FIGURE 2:**
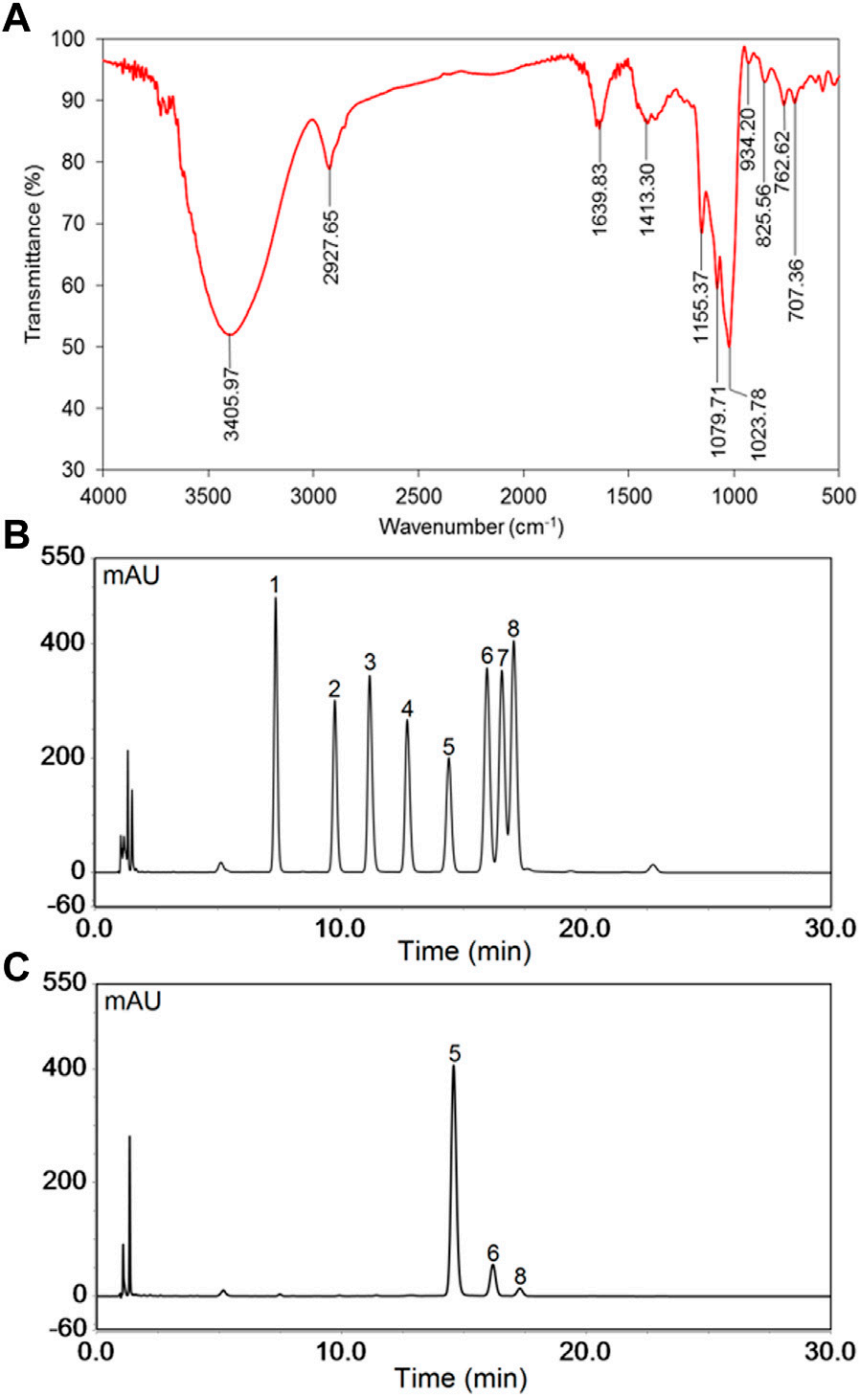
The FT-IR spectrum and monosaccharide composition of PNPB1. **(A)** FT-IR spectrum of PNPB1. **(B)** The HPLC of the derivatives of 8 standard monosaccharides. **(C)** The HPLC of the derivatives of PNPB1. (Peak identities: 1-Man; 2-Rha; 3-GlcA; 4-GalA; 5-Glc; 6-Gal; 7-Xyl; 8-Ara).

The FT-IR spectrum of PNPB1 displayed the characteristic peaks of polysaccharide, as shown in Figure 2A. The results of FT-IR spectrum indicated that the broad band at 3,200–3,600 cm^−1^ was attributed to the stretching vibration of O-H, and the band at 2,927.65 cm^−1^ was the stretching vibration of C-H. The absorption peaks at 1,639.83 and 1,413.30 cm^−1^ were assigned to the stretching vibration of carboxylic carbonyl group and C-O, respectively, indicating the presence of uronic acid in PNPB1 (Huang et al., 2016; Wang et al., 2017b). In addition, the peak at 825.56 cm^−1^ implied the existence of α-type glycosidic linkages in polysaccharide structure (Li and Shah, 2014).

### Monosaccharide composition analysis

The monosaccharide composition was determined by comparing the retention time of PMP-derived polysaccharide sample with those of standard monosaccharides in HPLC chromatograms. As illustrated in Figures 2B, C, the results indicated that PNPB1 consisted of Glc (88.2%), Gal (9.0%), Ara (2.4%) and trace GlcA. The major monosaccharides of PNPB1 were Glc and Gal, accounting for 97.2% of all monosaccharides, which suggested it mainly consisted of neutral polysaccharides. According to the reported data, the neutral monosaccharides from notoginseng were mainly composed of Glc, with less Gal, Ara and Rha, whereas acidic ones typically consisted of GlcA, Gal, Ara and Rha. Chan et al. reported a neutral polysaccharide fraction (PNPN) containing Glc (82.9%), Gal (12.3%) and Ara (2.3%) but failed to conduct further fractionation. In view of the similarity of sugar composition and proportion, we speculate that PNPB1 may be one of the components of the fraction (Chan et al., 2020).

### Methylation and GC-MS analysis

Methylation followed by GC–MS analysis was performed to determine the linkage patterns of the polysaccharide. The methylated alditol acetates of PNPB1 detected by GC-MS revealed that had a complex structure with seven types of linkages (Table 1). The presence of derivatives were identified as 2,3-Me_2_-Araf, 2,3,4,6-Me_4_-Glcp, 2,3,4-Me_3_-Glcp, 2,3,4-Me_3_-Galp, 2,3,6-Me_3_-Glcp, 2,3,6-Me_3_-Galp, and 2,3-Me_2_-Glcp in the molar ratios of 1.1:10.6:2.4:67.4:9.5:2.7:6.2 which suggested that PNPB1 was mainly composed of 1,4-Glcp, 1,4,6-Glcp and 1,6-Galp as the backbone and the molar ratio of these residues agreed overall with the monosaccharide composition.

**TABLE 1:** Methylation analysis by GC-MS of PNPB1.

No.	Methylated sugars	Linkage pattern	Molar ratio (%)
1	2,3-Me2-Araf	→5-Araf-1→	1.1
2	2,3,4,6-Me4-Glcp	Glcp-1→	10.6
3	2,3,4-Me3-Glcp	→6-Glcp-1→	2.4
4	2,3,6-Me3-Glcp	→4-Glcp-1→	67.4
5	2,3,4-Me3-Galp	→6-Galp-1→	9.5
6	2,3,6-Me3-Galp	→4-Galp-1→	2.7
7	2,3-Me2-Glcp	→4,6-Glcp-1→	6.2

### NMR analysis

**FIGURE 3:**
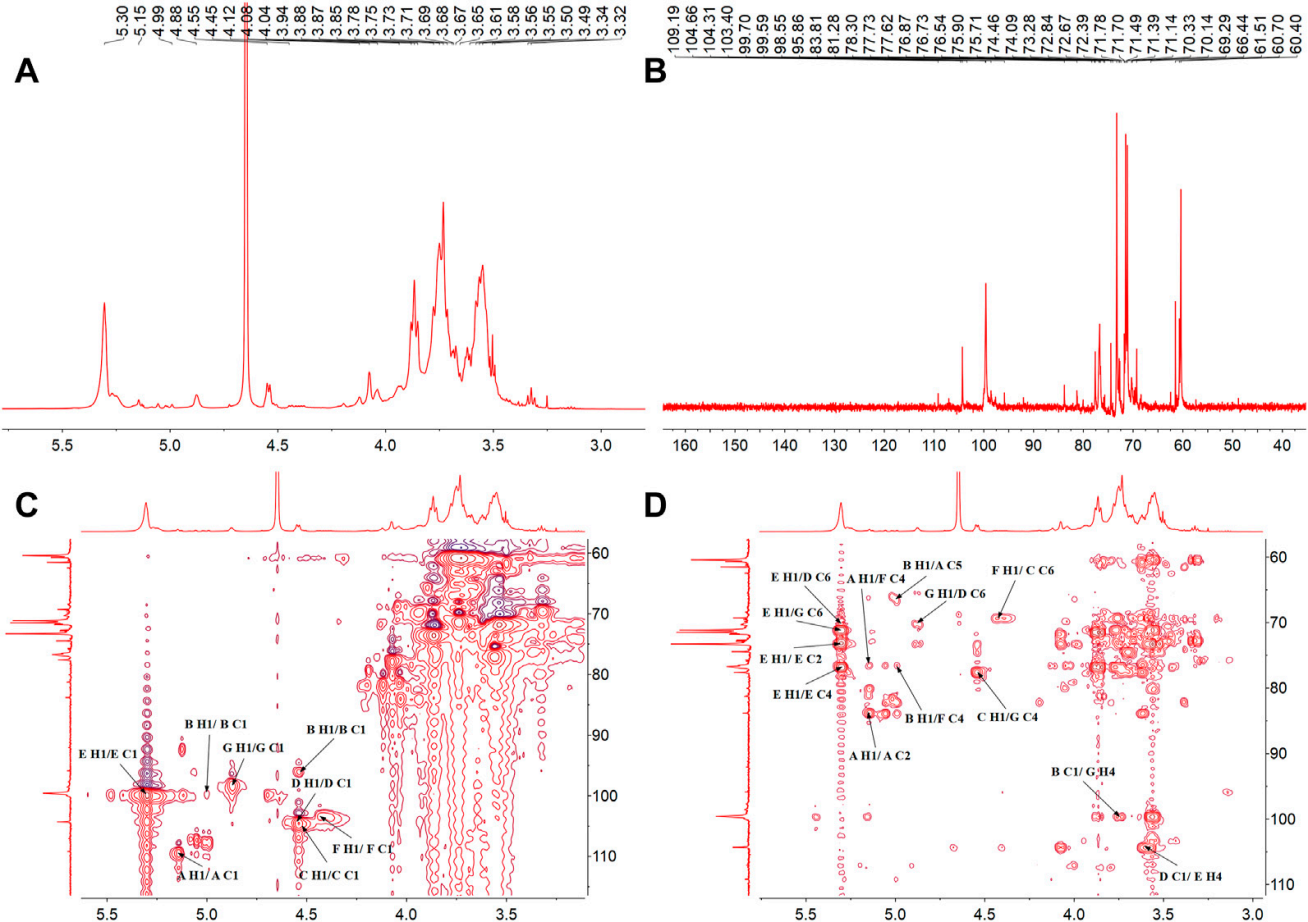
The NMR spectra of PNPB1. **(A)** ^1^H-NMR spectrum. **(B)** ^13^C-NMR spectrum. **(C)** HSQC spectrum. **(D)** HMBC spectrum.

The structure characteristics of the polysaccharide including sugar unit sequences and linkage patterns were further elucidated by 1D and 2D NMR spectra, including ^1^H-NMR, ^13^C-NMR, HSQC and HMBC. The signals of PNPB1 were in the regions ranging from 3.32 to 5.30 ppm in ^1^H-NMR and 60.40–109.19 ppm in ^13^C-NMR (Figures 3A, B). Compared with the 1H-NMR, 13C-NMR, HSQC and HMBC spectrum (Figures 3C, D) with the reported NMR data (Chen et al., 2017b; Wang et al., 2018; Feng et al., 2019; Ru et al., 2019; Wang et al., 2019), chemical shifts of each proton and carbon of every residue in PNPB1 were assigned in Table 2.

**TABLE 2:** Chemical shifts assignment of 1H and 13C-NMR spectrum of PNPB1.

No.	Sugar residue	Nucleus	Chemical shift (ppm)					
			1	2	3	4	5	6
A	→5)-α-Araf-(1→	^1^H	5.15	4.08	3.87	3.94	3.85	
		^13^C	109.19	81.27	78.30	83.81	66.44	
B	β-Glcp-(1→	^1^H	4.99	3.67	3.49	4.04	3.56	4.08
		^13^C	99.70	74.78	74.17	71.61	75.22	60.70
C	→6)-β-Glcp-(1→	^1^H	4.55	3.49	3.88	3.34	3.61	3.88
		^13^C	104.66	79.90	76.54	70.30	73.28	69.29
D	→6)-β-Galp-(1→	^1^H	4.54	3.68	3.88	3.65	4.12	3.85
		^13^C	104.31	77.73	78.30	75.90	72.84	70.33
E	→4)- α-Glcp-(1→	^1^H	5.30	3.65	3.73	3.58	3.71	3.86
		^13^C	99.58	73.38	72.38	76.77	73.67	60.4
F	→4)-β-Galp-(1→	^1^H	4.45	3.32	3.50	4.04	3.71	3.94
		^13^C	103.40	74.92	75.90	76.40	71.39	61.51
G	→4,6)-β-Glcp-(1→	^1^H	4.88	3.49	3.62	3.71	3.75	3.88
		^13^C	98.55	72.66	74.46	77.62	72.39	70.14

**FIGURE 4:**
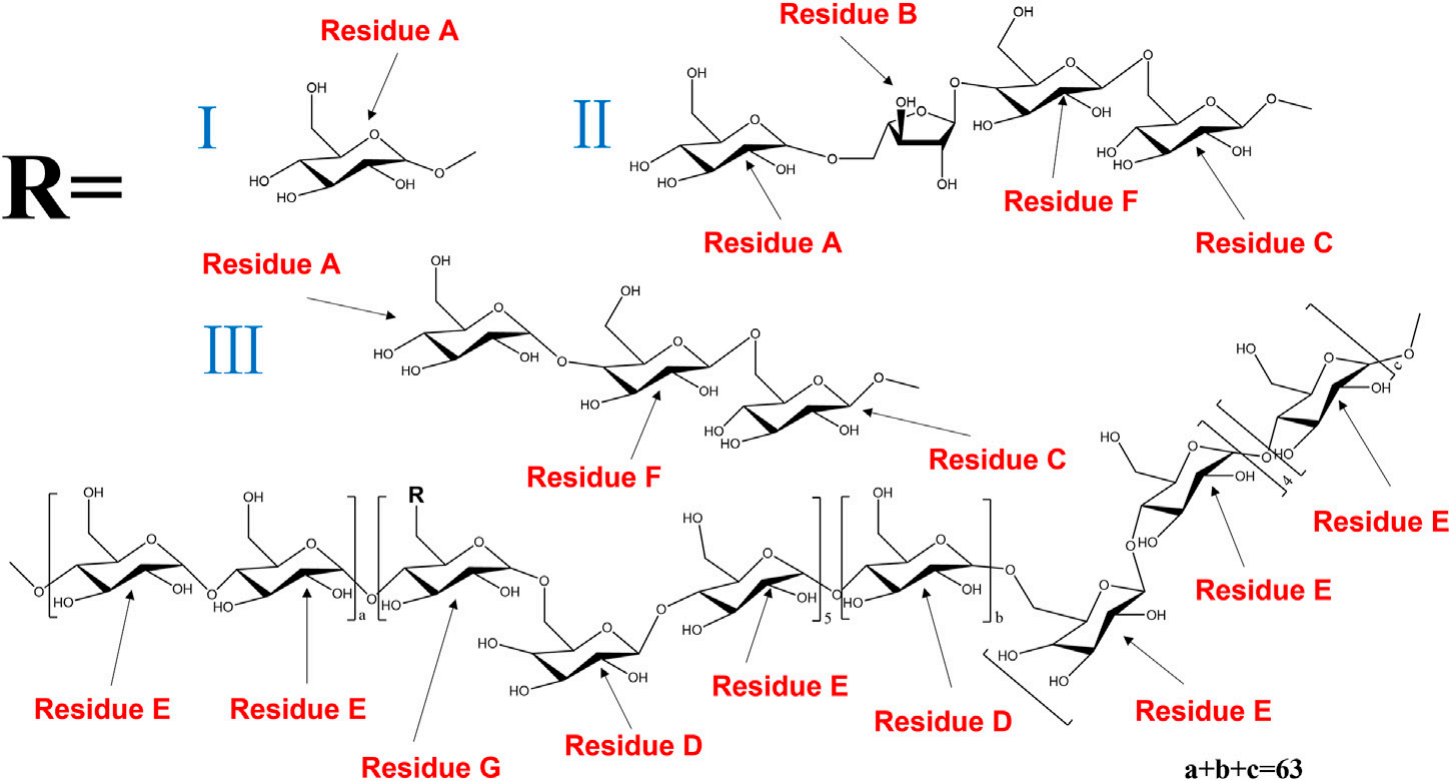
The possible structures of the repeating units in PNPB1.

The anomeric proton and carbon signals at δH5.15/δC109.19, δH4.99/δC99.70, δ4.55/104.60, δH4.55/δC104.31, δH5.30/δC99.58, δH4.45/δC103.40, and δH4.87/δC98.55 were assigned as A, B, C, D, E, F and G for α-Araf-(1→, β-Glcp-(1→, →6)-β-Glcp- (1→, →6)-β-Galp-(1→, →4)-α-Glcp-(1→, →4)-β-Galp-(1→, →4,6)-β-Glcp-(1→, respectively. Based on the HMBC spectrum, signals at δ5.30/70.33 (EH1/DC6), δ4.55/70.14 (DH1/GC6), δ5.30/76.77 (EH1/EC4), δ5.15/76.40 (AH1/FC4), δ4.99/66.44 (BH1/AC5), δ4.55/70.33 (CH1/DC6), δ4.55/76.40 (DH1/FC4), δ4.45/69.29 (FH1/CC6), δ4.55/77.62 (CH1/GC4), δ99.70/3.71 (BC1/GH4) and δ4.55/76.77 (DC1/EH4) were presented as the linkages of residue E O-1 with residue D C-6, residue D O-1 with residue G C-6, residue E O-1 with residue E C-4, residue A O-1 with residue F C-4, residue B O-1 with residue A C-5, residue C O-1 with residue D C-6, residue D O-1 with residue F C-4, residue F O-1 with residue C C-6, residue C O-1 with residue G C-4, residue G O-4 with residue B C-1 and residue E O-4 with residue D C-1. Based on the results of monosaccharide composition, methylation analysis and NMR analysis, the possible repetitive structure unit of PNPB1 was deduced and depicted in Figure 4.

### Morphological properties of PNPB1

**FIGURE 5:**
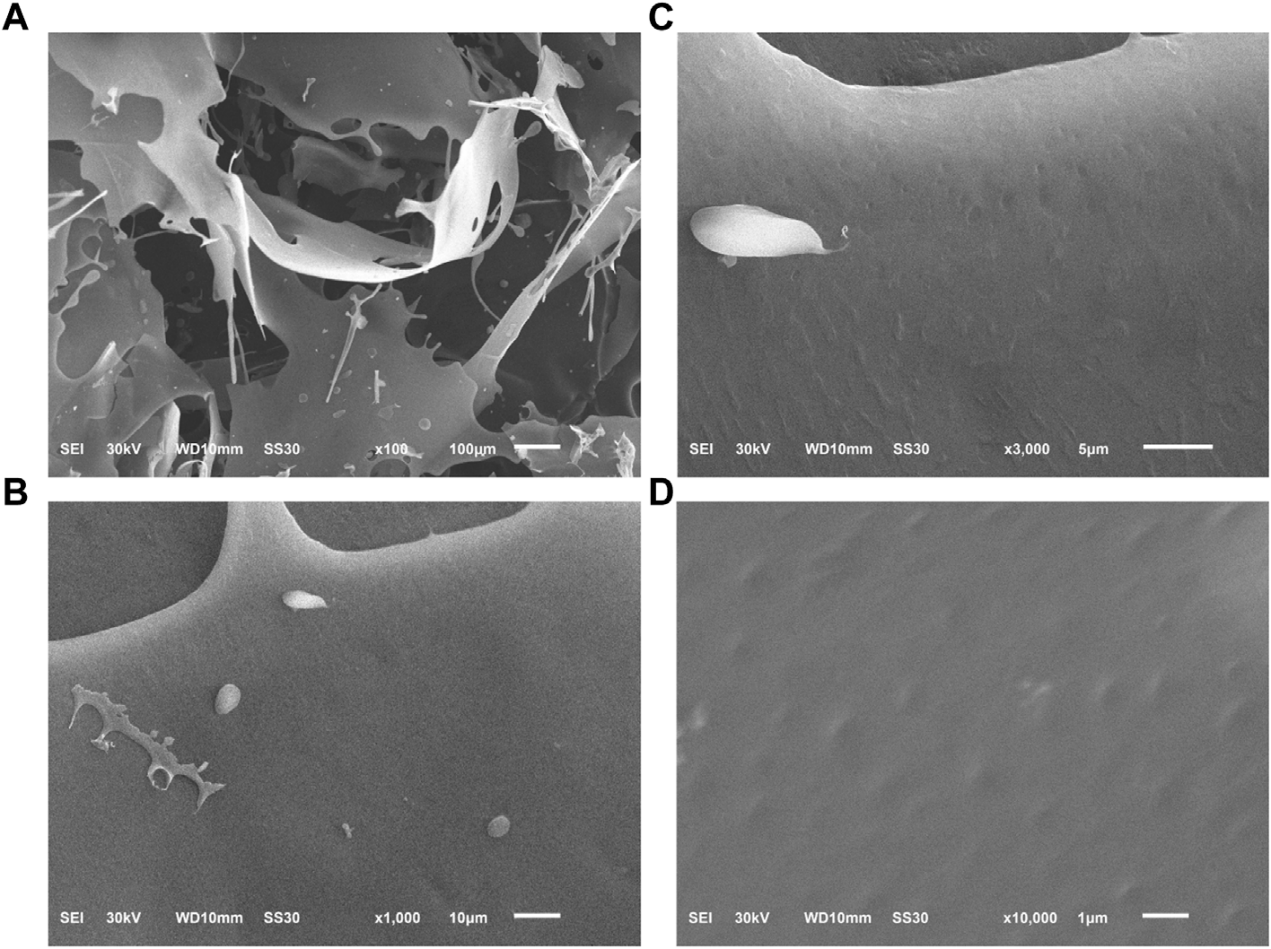
The microscopic morphology of PNPB1 by SEM. [**(A)**: × 100; **(B)** × 1,000; **(C)** × 3,000; **(D)** × 10,000].

The sample was morphologically characterized by SEM as a laminar structure with smooth surface under low magnification (×100), while the microstructure up to 3,000 times magnification revealed the arrays of irregular dotted pits in the surface (Figure 5).

### Immunomodulatory activity of PNPB1

**FIGURE 6:**
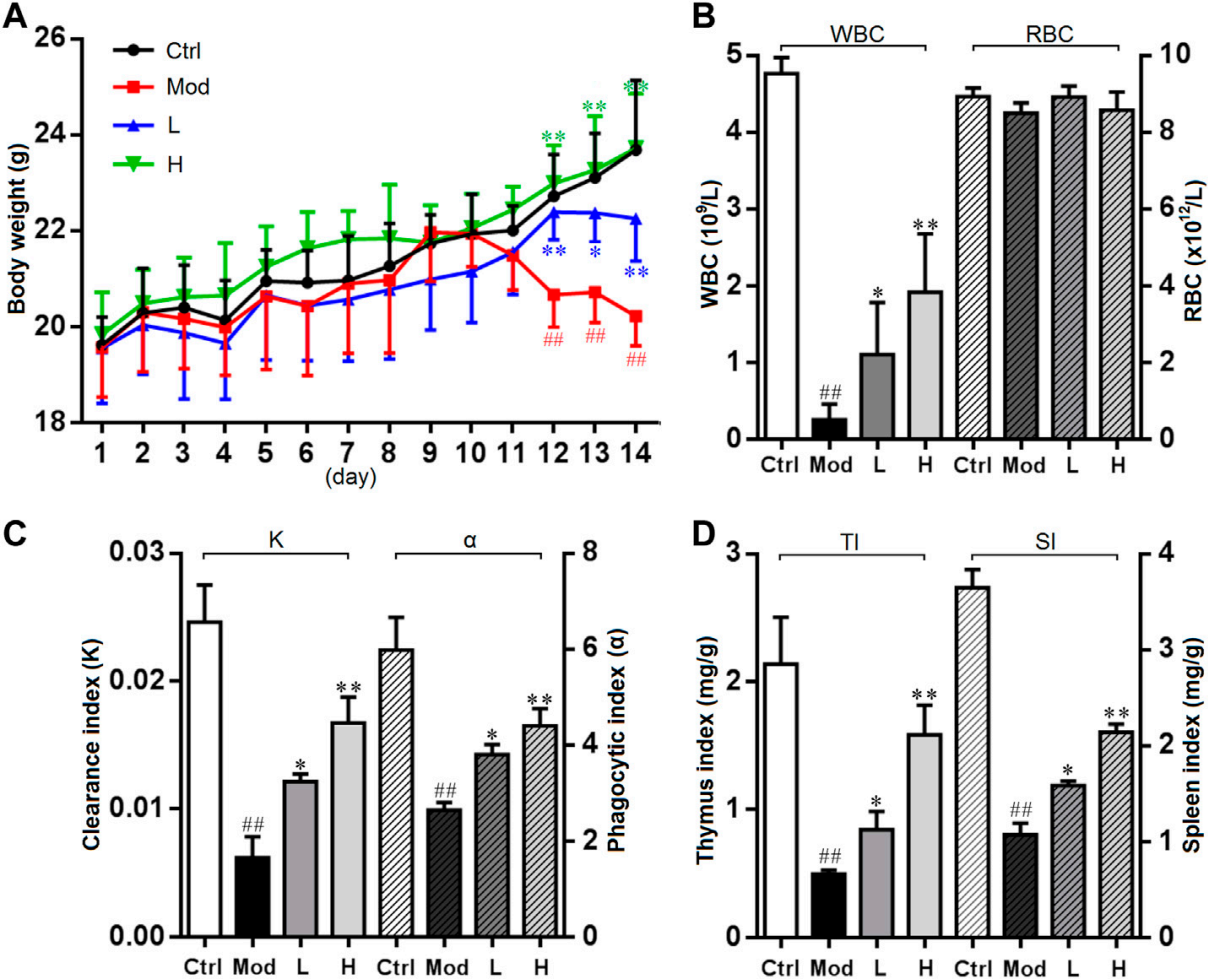
Immunomodulatory activity of PNPB1 in mice. **(A)** Effects of the PNPB1 on body weight. **(B)** WBC and RBC counts. **(C)** Clearance/phagocytic index. **(D)** Thymus/spleen index. (^##^ *p* < 0.01 vs. control group, *p < 0.05 and **p < 0.01 vs. model group).

Being an anti-mitotic and apoptosis inducing agent, CTX has strong immunosuppressive effect and is often used for the preparation of immunosuppressive model, which causes body weight loss and bone marrow suppression in animals. Previous studies have found some notoginseng polysaccharides, such as NPPN, a neutral polysaccharide (232.50 kDa), played a bone marrow protective role through increasing peripheral blood counts, relieving cell cycle arrest, and decreasing the apoptosis rate of bone marrow cells in myelosuppression mice induced by CTX (Liu et al., 2022a). By contrast, the polysaccharide isolated in the present study conferred a much more potent protection against murine myelosuppression. As shown in Figure 6A, the body weight of the mice was significantly decreased after treatment with CTX, whereas the trend of weight loss was notably mitigated in the low and high dose of PNPB1 groups, indicating that PNPB1 alleviated the immunological injury caused by CTX. Figure 6B showed the peripheral blood parameters including WBC and RBC of each group. After treatment with PNPB1, the leukocyte counts in model group fell to less than 0.2 × 10^9^/L, while those in low and high PNPB1 group markedly increased to 1.1 × 10^9^/L and 1.9 × 10^9^/L, respectively (*p* < 0.05). In addition, the peripheral blood cell levels of mice were unaffected by CTX or PNPB1 treatment and each group displayed no statistical difference.

As shown in Figure 6C, there was statistically significant difference in carbonic particle clearance index and macrophage phagocytose index, while the indices of all treated groups were higher (*p* < 0.05) than those of model group and showed a dose-dependent manner. These results suggested that PNPB1 could increase macrophage phagocytosis to a normal level in immunosuppressed mice, which was consistent with the results of Liu et al. (2022b). The thymus and spleen are the main immune organs of the body, and changes in their weight can reflect the stimulating effect of tested drugs on the immune system. As shown in Figure 6D, compared with the model group, the thymus index and spleen index of the low-dose and high-dose PNPB1 groups were significantly increased (*p* < 0.05) in a dose-dependent manner, which were better than the corresponding immune effects reported for other notoginseng polysaccharides (Liu et al., 2021).

**FIGURE 7:**
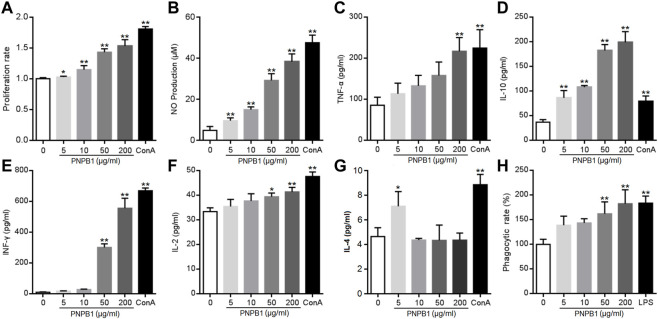
**(A)** Effects of the PNPB1 on *in vitro* splenocyte proliferation. **(B–G)** Production of NO, TNF-a, IFN-γ, IL-2, IL-4, IL-10 by murine splenocytes. **(H)** Phagocytosis rate of neutral red in macrophage. (*p < 0.05 and **p < 0.01 vs. control group).

Lymphocytes are the main immune effector cells involved in adaptive immune system, and their proliferation and transformation can reflect the immune function of the body. T lymphocytes mainly mediate cellular responses and can undergo mitosis after incubation with ConA *in vitro*, while B lymphocytes initiate humoral immune response and proliferate by the stimulus LPS (Feng et al., 2016). As spleen is the main organ for T and B lymphocytes to settle, splenocyte proliferation rate is known as the most direct indicator of cellular immune ability (Chen et al., 2007). It was revealed that crude polysaccharide from notoginseng could promote murine spleen lymphocyte proliferation and increase the CD3^+^, CD4^+^ T-cell population in the peripheral blood of tumor-bearing mice, suggesting that PNP enhanced cellular immunity *in vitro and in vivo* (Abdin and Hasby, 2014; Li et al., 2016). In this study, PNPB1 dose-dependently increased lymphocyte proliferation at the concentration of 5–200 μg/mL, as presented in Figure 7A. The polysaccharide displayed significant induction on splenocyte proliferation even at the lowest dose (5 μg/mL), and induced the proliferation rate up to 53% at the dose of 200 μg/mL compared to the untreated cells.

Besides the stimulation on the proliferation of immune cells, promoting the cytokine secretion is another important way for polysaccharides to exert immune function. Cytokines are small molecular proteins or polypeptides mainly secreted by activated immune cells, which are vital for lymphocytes to mediate immune response. Naive CD4^+^ T-cell differentiate into Th1 or Th2 cells under antigen stimulation, which mediate the cellular and humoral immune responses, respectively. Th1 cells are characterized by the secretion of TNF-α, IFN-γ, IL-2 and IL-12, while Th2 cells produce IL-4, IL-5, IL-10, and IL-13. The released cytokines modulate the functions of Th cellular immune response and cross-inhibition of Th1 and Th2 cells (Zhu et al., 2006). Liu et al. observed that a homogeneous polysaccharide PNPS-0.3 (76.6 kDa) extracted from notoginseng induced significantly the levels of TNF-α, IFN-γ and IL-12 secretion in dendritic cells at 12.5–50 μg/mL, indicating that PNP could regulate immunity by enhanced cytokine secretion (Liu et al., 2020). Current experiment revealed that PNPB1 (5–200 μg/mL) treatment induced a significant augmentation in cytokine secretion. As shown in Figures 7B–F, PNPB1 enhanced TNF-α, IFN-γ and IL-2 secretion in a dose-dependent manner. The concentration of TNF-α showed a rising trend with the treatment of PNPB1 at 5–50 μg/mL, and increased to a level comparable to ConA group when the dose reached 200 μg/mL (*p* < 0.01). The productions of IFN-γ and IL-2 were also increased remarkably with PNPB1 concentrations up to 50 μg/mL (*p* < 0.05), especially the secretions of IFN-γ, which were elevated to 57 and 68 times those in the control group after treatment with PNPB1 at 50 and 200 μg/mL, respectively (*p* < 0.01). However, PNPB1 showed little impact on the IL-4 production in splenocytes over 5 μg/mL, but strongly elevated the concentration of IL-10 even at the lowest concentration (5 μg/mL), indicating that PNPB1 could enhance both Th1 and Th2 cytokines production with varying degrees *in vitro*, and has the potential to alter Th1/Th2 immune balance.

NO, a cytotoxic substance secreted by activated immune cells to kill pathogens and tumor cells, is also an important mediator of intercellular information transmission that can enhance the nonspecific immune function through complex interaction with various cytokines (Bogdan et al., 2000). As shown in Figure 7B, the NO content increased markedly from 4.93 μM in the control group to 9.70, 15.00, 29.28, and 38.41 μM in the presence of PNPB1 concentrations of 5–200 μg/mL, and the maximum yield of NO was up to 80% of the positive control (5 μg/mL ConA). Macrophages are important regulatory and effector cells of immunity, which control the inflammatory and immune response by phagocytizing and killing microorganisms, antigen presentation and cytokine secretion. Evidences have proved that the immunoregulation of plant polysaccharides is closely related to the activation of macrophages (Yu et al., 2020). A notoginseng polysaccharide Pnp (78.6 kDa, 31.25–500 μg/mL) was found to promote the secretion of IL-6, TNF-α and NO, as well as stimulate the phagocytosis of RAW 264.7 cells in a dose-dependent manner (Yang et al., 2023). To ascertain the potential of PNPB1 on activating macrophages, the phagocytosis was investigated by the neutral red up-take bioassay at PNPB1-treated macrophages. The results demonstrated that the phagocytosis was significantly enhanced with the increased concentration of PNPB1. The polysaccharide of 200 μg/mL obtained a comparable response with LPS (1 μg/mL) by boosting the phagocytosis rate to 183% (Figure 7G). Although higher doses were required for the polysaccharide to play a significant effect, it exhibited a strong activity while considering its appreciable molecular weight.

**FIGURE 8:**
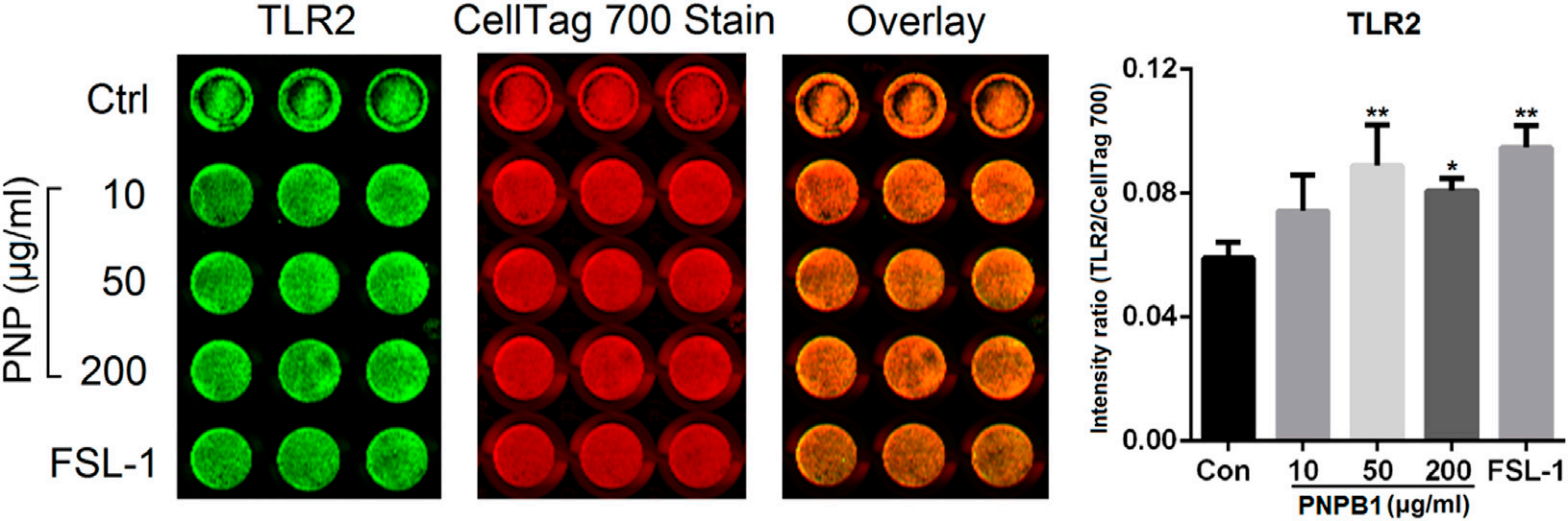
ICW quantitation of TLR2 protein expression in macrophage regulated by PNPB1. (*p < 0.05 and **p < 0.01 vs. control group).

Toll like receptor 2/4 (TLR2/4) are considered to play important roles in the innate immune response and involved in the immediate immune response induced by polysaccharides, which can be directly induced by stimulus and mediate the secretion of NO, TNF, IFNs, ILs and other substances from immunocytes. The receptor affinity of polysaccharides is closely related to their structure, for instance, glucose and mannose fractions in polysaccharides are prone to be recognized by TLR2, whereas carboxylic or acetyl groups are essential in TLR4 binding. The above mentioned polysaccharide Pnp can bind to TLR2, while PNPS-0.3 might primarily bind to TLR4 due to the abundant carboxylic group in GalA fractions (Liu et al., 2020; Yang et al., 2023). The protein expression of TLR2/4 in phagocytes affected by PNPB1 was analyzed by ICW and the result showed that TLR2 protein level were upregulated after the treatment of polysaccharide at the dose of 50 and 200 μg/mL, which was comparable with the effect of TLR2 agonist FSL-1 at 1 μg/mL (Figure 8). However, TLR4 protein level was unaffected by the treatment of PNPB1 (data not shown), implying that the PNPB1 immunoenhancement effect might involve the protein regulation of TLR2 but not TLR4.

## Conclusion

A novel water-soluble polysaccharide PNPB1 was isolated from notoginseng. Its chemical structure was characterised by FT-IR, GC–MS, 1D and 2D NMR spectroscopic techniques and the immunomodulatory activities were studied *in vivo* and *in vitro*. PNPB1 possesses a high molecular weight up to 930 kDa with a complex structure involving three kinds of monosaccharides and seven types of linkages. The present work demonstrated that PNPB1 could significantly enhance body weight, improve thymus and spleen indices and increase the WBC in immunosuppressive mice. The polysaccharide might mediate immunopotentiation through the enhancement of splenocyte proliferation, secretion of TNF-α, IFN-γ, IL-2, IL-10 as well as phagocytosis of macrophages, and involve the protein regulation of TLR2, which could be used as a potential immunological response modifier.

## Data Availability

The datasets presented in this study can be found in online repositories. The names of the repository/repositories and accession number(s) can be found in the article/Supplementary Material.
